# Changes in the Bacterial Diversity of Human Milk during Late Lactation Period (Weeks 21 to 48)

**DOI:** 10.3390/foods9091184

**Published:** 2020-08-27

**Authors:** Wendy Marin-Gómez, Mᵃ José Grande, Rubén Pérez-Pulido, Antonio Galvez, Rosario Lucas

**Affiliations:** Microbiology Division, Department of Health Sciences, Faculty of Experimental Sciences, University of Jaén, 23071 Jaén, Spain; wendymarin@gmail.com (W.M.-G.); mjgrande@ujaen.es (M.J.G.); rppulido@ujaen.es (R.P.-P.); rlucas@ujaen.es (R.L.)

**Keywords:** breast milk, biodiversity, lactic acid bacteria, late lactation, metagenomics

## Abstract

Breast milk from a single mother was collected during a 28-week lactation period. Bacterial diversity was studied by amplicon sequencing analysis of the V3-V4 variable region of the 16S rRNA gene. *Firmicutes* and *Proteobacteria* were the main phyla detected in the milk samples, followed by *Actinobacteria* and *Bacteroidetes*. The proportion of *Firmicutes* to *Proteobacteria* changed considerably depending on the sampling week. A total of 411 genera or higher taxons were detected in the set of samples. Genus *Streptococcus* was detected during the 28-week sampling period, at relative abundances between 2.0% and 68.8%, and it was the most abundant group in 14 of the samples. *Carnobacterium* and *Lactobacillus* had low relative abundances. At the genus level, bacterial diversity changed considerably at certain weeks within the studied period. The weeks or periods with lowest relative abundance of *Streptococcus* had more diverse bacterial compositions including genera belonging to *Proteobacteria* that were poorly represented in the rest of the samples.

## 1. Introduction

Human milk is considered to be an important source of bacteria for the newborn. Many of these bacteria may be human commensals or have potential probiotic effects [1]. Lactic acid bacteria, such as *Lactobacillus fermentum*, *L. gasseri*, *L. rhamnosus*, isolated from human breast milk, can be regarded as potential probiotic bacteria [2,3,4]. Previous studies have suggested that commensal coagulase-negative staphylococci and viridans streptococci found in breast milk can reduce the acquisition of undesired pathogens by infants exposed to hospital environments [5]. In addition, some of the bacterial strains found in human milk may have a large potential to improve the mother’s health [6]. Furthermore, bacteria ingested during breastfeeding contribute to the development of the infant gut microbiome [7]. The benefits of breastfeeding also extend to a reduction of respiratory and gastrointestinal tract infections and to a correct education of the immune system, with a concomitant reduction of the risks to develop several diseases such as obesity, diabetes, or inflammatory bowel diseases [7,8,9].

In addition to classical studies based on isolation and identification of bacteria from human milk [5], culture-independent studies have provided a large amount of information on the human milk microbiota. The culture-independent approaches based on amplification and sequencing of variable regions within the 16S rDNA gene, allow the detection in a single step of both aerobic and anaerobic bacteria as well as bacteria that, in spite of being in a low proportion in the population, may play significant roles. Recent review papers have summarized the major findings of previous studies on the microbiota from human milk samples based on culture-independent approaches [10,11]. Many of the studies have focused on the influence of different factors such as the mother’s diet and health status, maternal age, child delivery method, probiotic use, HIV infection, administration of antibiotics or collection/feeding method, and involve samples from several subjects. Such studies led to the proposal of a core microbiota for the human milk or at least a list of the bacterial genera most frequently found [11]. Nevertheless, the influence of the lactation stage has been studied to a much less extent. Cabrera-Rubio et al. [12] analyzed the milk samples from 18 women at three sampling points, e.g., within 2 days after mothers gave birth in the maternity hospital (colostrum) and at 1 and 6 months after delivery at home. The main results obtained after 16S rRNA gene amplification and pyrosequencing indicated that the human milk microbiome changes over lactation: *Weissella*, *Leuconostoc*, *Staphylococcus*, *Streptococcus*, and *Lactococcus* were predominant in colostrum samples, whereas in 1- and 6-month milk samples, the typical inhabitants of the oral cavity (e.g., *Veillonella*, *Leptotrichia*, and *Prevotella*) increased significantly. Khodayar-Pardo et al. [13] applied quantitative polymerase chain reaction (PCR) to study the microbial composition of milk samples collected from 322 mothers within the first month of exclusive breastfeeding and reported that total bacteria, *Bifidobacterium* and *Enterococcus* spp. counts, increased throughout the lactation period. Most of these studies, however, do not report individual variations in the microbiota during the lactation period. Nevertheless, it is suspected that different changes may occur at the individual level due to different factors. The aim of the present study was to investigate the microbiota during the mid-to-late lactation period in breast milk from a single mother and to analyze possible changes in bacterial diversity during the period.

## 2. Materials and Methods

### 2.1. Sample Collection

Written informed consent was obtained, in accordance with the Declaration of Helsinki. Samples were taken from a single 38-year-old Latin American female donor, suffering from asthma and overweight, during a lactating period between weeks 21 and 48, inclusive, after cesarean delivery. Samples were taken three times in the day before baby lactation. The hands of the volunteer were cleaned with soap and covered with sterile gloves. Then, the nipples and surrounding areola were cleaned with cotton soaked with 70% ethanol. The first drops of milk were discarded, and then 5–7 mL of milk was extracted from each breast manually using a sterile manual pump (Philips Avent SCF330/20; Philips Ibérica, Madrid, Spain). The milk was transferred to sterile falcon test tubes, stored at 4 °C, and transported on ice to the laboratory within the next 12 h, where it was stored at −20 °C until analysis.

### 2.2. DNA Extraction

Thawed milk samples from the same day were mixed thoroughly and centrifuged at 16,000× *g* for 7 min in a refrigerated centrifuge 5424 R (Eppendorf, Corp., Hamburg, Germany). After removal of the supernatants, total DNA from the remaining pellets was extracted with a QIAamp Stool DNA Fast Mini Kit (Qiagen, Madrid, Spain) following the manufacturer instructions. The quality and quantity of the extracted DNA was determined by QuantiFluor^®^ ONE dsDNA system (Promega, Madison, WI, USA). The DNA was stored at −20 °C until analysis.

### 2.3. DNA Sequencing and Analysis

The 16S rDNA V3-V4 regions were amplified following the Illumina Metagenomics Sequencing Library Preparation protocol (Illumina, Inc., San Diego, CA, USA). Illumina adapter overhang nucleotide sequences were added to the gene-specific sequences. The following 16S rDNA gene amplicon PCR primer sequences were used: forward primer: 5′TCGTCGGCAGCGTCAGATGTGTATAAGAGACAGCCTACGGGNGGCWGCAG; reverse primer: 5′GTCTCGTGGGCTCGGAGATGTGTATAAGAGACAGGACTACHVGGGTATCTAATCC [14]. Microbial genomic DNA (5 ng/μL in 10 mM Tris pH 8.5) was used to initiate the protocol. After 16S rDNA gene amplification, the multiplexing step was performed using Nextera XT Index Kit (Illumina). Then, 1 μL of the PCR product was run on a Bioanalyzer DNA 1000 chip to verify the size (expected size ~550 bp). After size verification, the libraries were sequenced using a 2 × 300 pb paired-end run on a MiSeq Sequencer according to the manufacturer’s instructions (Illumina). Quality assessment was performed by the use of prinseq-lite program [15]. The sequence data were analyzed using qiime2 pipeline [16]. Denoising, paired-ends joining, and chimera depletion were performed starting from paired ends data using DADA2 pipeline [17]. Taxonomic affiliations were assigned using the Naive Bayesian classifier integrated in quiime2 plugins and the SILVA_release_132 database. Statistical analysis was carried out with SPSS software version 24 (IBM Corp., Foster City, CA, USA).

## 3. Results

### 3.1. Characteristics of Sequence Reads

The numbers of reads assigned to operational taxonomic units (OTUs) and the alpha diversity indicators are shown in Table 1. The number of assigned reads ranged from 140 to 1,375,641. A few samples yielded very low numbers of reads (e.g., samples corresponding to weeks 11, 15, 19, and 26 of the sampling period) and yielded a very low number of observations (between 3 and 9). In addition, samples 11 and 26 showed an abnormal bacterial composition. Therefore, these samples were excluded from the analysis.

### 3.2. Bacterial Diversity in Breast Milk Samples

**Figure 1:**
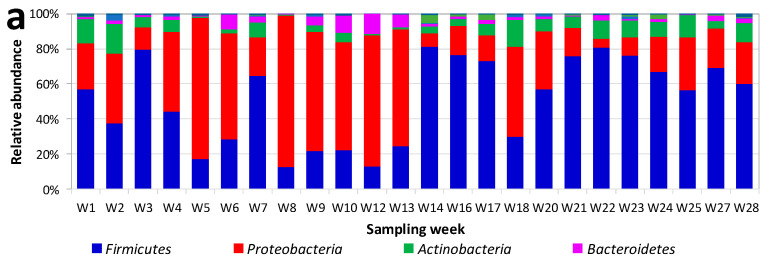

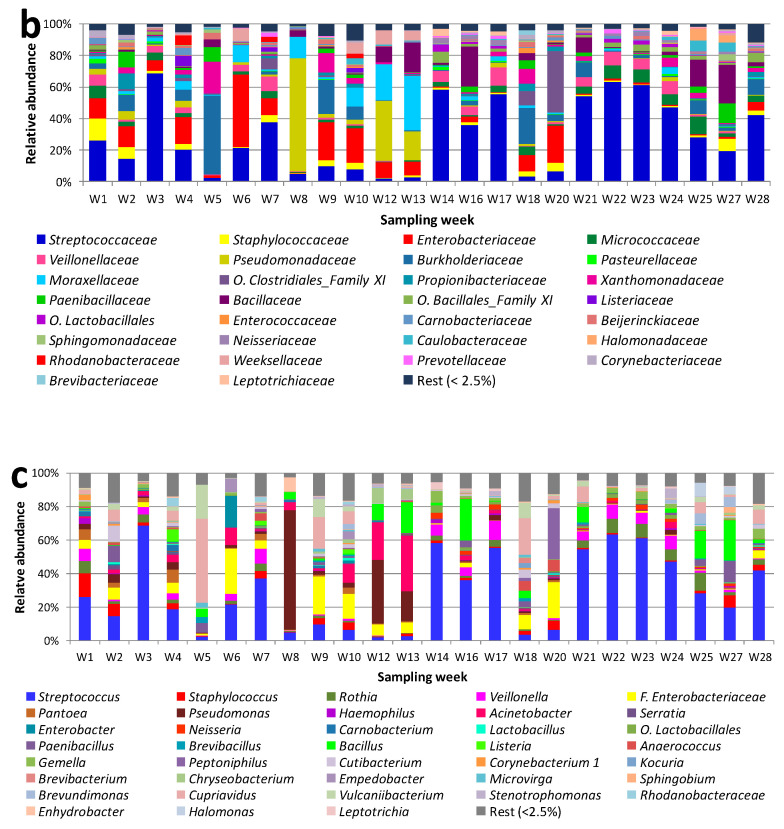
Bacterial diversity of breast milk samples at Phylum (**a**), Family (**b**) and Genus (**c**) levels. The different sampling weeks (W) are represented.

*Firmicutes* and *Proteobacteria* were the main phyla detected in the milk samples (Figure 1a). The proportion of *Firmicutes* to *Proteobacteria* changed considerably depending on the sampling week. *Firmicutes* were most abundant in samples from weeks 1, 3, 7, 14–17, 20–25, and 27–28 of the sampling period. However, *Proteobacteria* were predominant in the rest of the samples. *Actinobacteria* were the third most important group in most of the samples, followed by *Bacteroidetes*. *Bacteroidetes* had higher relative abundances than *Actinobacteria* in samples corresponding to weeks 6 and 10–13. The above-mentioned phyla represented between 98.0% and 99.8% of the OTUs.

The 30 families that had relative abundances ≥ 2.5% are shown in Figure 1b. These covered between 88.1 and 98.2 of OTUs. *Firmicutes* were represented mainly by Fam. *Streptococcaceae*. This group was found at relative abundances in a range from 2.0% to 68.8%. The following families were represented in many of the samples: *Staphylococcaceae*, *Bacillaceae*, *Paenibacillaceae*, and *Veillonellaceae*. Members of O. *Clostridiales* Family XI were detected only in a few of the samples. Unidentified members of O. *Lactobacillales* and *Carnobacteriaceae* were also relevant in some samples, with relative abundances ranging from ca. 1.5% to ca. 4.5%. *Lactobacillaceae* only were represented in two samples (W2, W10), with relative abundances between 1.0% and 1.2%. *Enterobacteriaceae* were highly represented in most of the samples, ranking sometimes in first position in relative abundance. Members of *Pseudomonadaceae*, *Moraxellaceae*, and *Xanthomonadaceae* were also relevant groups among the *Proteobacteria*. *Microccaceae* and *Weeksellaceae* were the main representatives among *Actinobacteria* and *Bacteroidetes*, respectively.

**Figure 2:**
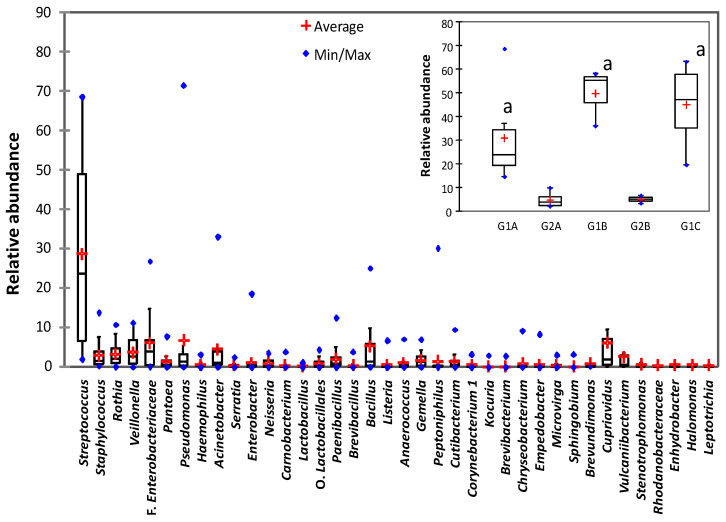
Box-plot representation of the relative abundances of the 38 main bacterial genera detected in the milk samples. Insert: Box-plot representation of the relative abundances of gen. *Streptococcus* during the sampling period. Data were grouped by weeks according to relative abundance. Groups with high relative abundance: G1A (weeks 1–4, 6–7), G1B (weeks 14, 16–17), G1C (weeks 21–25, 27–28). Groups with low relative abundance: G2A (weeks 5, 8–10, 12–13), G2B (weeks 18, 20). ^a^ statistically significant differences (*p* < 0.05) with G2A.

The 38 genera with relative abundances ≥ 2.5% (representing between 81.7 and 97.5 of OTUs) are shown in Figure 1c. Genus *Streptococcus* was detected in all the samples (being the most abundant OTU in 14 samples), although there were large differences in its relative abundances between samples. For example, genus *Streptococcus* had relative abundances that were above 50% in samples from weeks 3, 14, 17, and 21–23. By contrast, the lowest relative abundances for this genus were detected in samples from weeks 5, 8–10, 12–13, 18, and 20. Box-plot representation of the relative abundances of the main genera across the samples (Figure 2) indicated that *Streptococcus* was the main genus in the milk (Figure 2). Data on the relative abundance of *Streptococcus* were clustered in three groups of high relative abundances with two intercalated periods of low relative abundances (Figure 2, insert) and then analyzed by univariate statistical analysis (ANOVA, Tukey’s test, Kruskal–Wallis, Dunn’s post hoc). The results revealed that the three main groups of samples (G1A, G1B, G1C) had significantly higher relative abundances (*p* < 0.05) than the low-abundance samples. Apparently, groups G1B and G1C had higher relative abundances than group G1A (which would suggest an increase in the relative abundance of *Streptococcus* by the end of the sampling period). However, the differences between the three groups were not statistically significant (*p* > 0.05) for any of the univariate analyses carried out.

Another genus represented in all breast milk samples was *Staphylococcus* (Figure 1c), with relative abundances from 0.3% to 14%. The lactic acid bacteria *Carnobacterium* and *Lactobacillus* had very low relative abundances. Aerobic endospore formers (*Paenibacillus*, *Brevibacillus*, and *Bacillus*) were detected in many of the samples, in some cases with high relative abundances. *Gemella* was also detected in many samples, and *Listeria* was detected in three samples (reaching 6.8% in one sample).

Most of the *Enterobacteriales* belonged to Fam. *Enterobacteriaceae* (Others). Members of the genera *Pantoea*, *Serratia*, and *Enterobacter* were also relevant in some samples (Figure 1c). Members of the genera *Acinetobacter*, *Haemophilus*, and *Neisseria* were detected in some samples. *Pseudomonas* had low relative abundances, except for three samples (weeks 8, 12, and 13). Several samples showed high relative abundances of members of family *Burkholderiaceae* (*Cupriavidus*), with remarkably high values at week 5. All samples with a high relative abundance of *Cupriavidus* also had higher relative abundances of *Vulcaniibacterium* (*Xanthomonadaceae*). Among *Actinobacteria*, the main representative was *Rothia* (with relative abundances between 3% and 10% in many samples).

**Figure 3:**
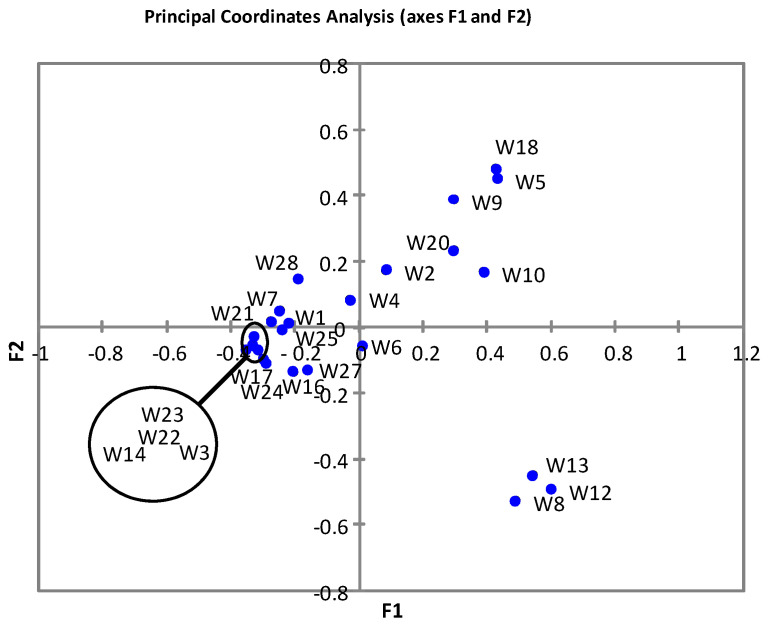
Principal coordinates analysis of breast milk samples taken at different weeks (W) during the sampling period.

PCoA (Figure 3) revealed a main cluster of samples (all of them having a mid-to high relative abundance of OTUs belonging to genus *Staphylococcus*), with at least two minor clusters (samples W8, W12, W13) characterized by a very low relative abundance of *Streptococcus* and high relative abundances of *Pseudomonas*/*Acinetobacter*/*Bacillus*, and samples W5, W9, W10, W18 (also having very low relative abundances of *Streptococcus* and higher relative abundances of *Cupriavidus*/*Vulcaniibacterium* together with other bacterial groups).

## 4. Discussion

Results from the present study provided information on the changes in bacterial diversity in human milk from a single individual during the late lactating period. By focusing on a single donor, the present study avoided the confounding effect of data from different subjects and allowed to follow this mother continuously. Although the data may be more difficult to analyze, the study revealed a sample variability that otherwise may be unnoticed in studies involving a compendium of samples. Many previous studies have addressed the microbial composition of human milk, but usually from a compendium of samples taken from different individuals and taken at early to mid lactating period. Zimmermann and Curtis [11] identified 44 studies investigating 3105 breast milk samples from 2655 women, and reported that the most frequently found genera were *Staphylococcus*, *Streptococcus*, *Lactobacillus*, *Pseudomonas*, *Bifidobacterium*, *Corynebacterium*, *Enterococcus*, *Acinetobacter*, *Rothia*, *Cutibacterium*, *Veillonella*, and *Bacteroides*. Another review paper reported that *Streptococcus* and *Staphylococcus* appear to be widely predominant in human milk without regard to differences in geographic location or analytic methods [10].

Results from the present study also identified main genera reported in previous studies (*Streptococcus*, *Staphylococcus*, *Pseudomonas*, *Acinetobacter*, *Rothia*, *Cutibacterium*, and *Veillonella*). Furthermore, the microbial composition of several samples from the present study resembled, in a certain way, that of the children salivary microbiome reported by other authors [18] (with the following common genera: *Streptococcus*, *Veillonella*, *Rothia*, *Leptotrichia*, *Haemophilus,* and *Neisseria*). These results would suggest colonization of mammary glands by bacteria from the baby’s mouth. As a matter of fact, human milk is considered to be colonized by bacteria from the mother’s gut and skin or the infant’s mouth [19,20].

*Streptococcus* was the predominant OTU in 14 of the samples. Remarkably, those samples with lower relative abundances of *Streptococcus* had different microbial compositions. While the sequence reads obtained in the present study only allowed identification at the genus level, a previous study reported the presence of the following species of *Streptococcus* in human milk: *S. mitis*, *S. infantis*, *S. cristatus*, *S. salivarius*, *S. mutans*, *S. sanguinis*, *S. gordonii*, and *S. sanguinosus* [21]. Streptococci may produce different types of antimicrobial substances including hydrogen peroxide, organic acids, and bacteriocins [22,23]. The obtained results would suggest an ecological role of *Streptococcus* in the control of microbial populations in breast milk. *Staphylococcus* was also detected in all breast milk samples, with relative abundances reaching up to 14% in one sample. It has been suggested that commensal coagulase-negative staphylococci and viridans streptococci from breast milk could reduce the acquisition of undesired pathogens by infants, especially when exposed to hospital environments [5,24].

Contrary to other studies, *Lactobacillus* had low relative abundance in the studied milk samples, and other related genera (*Enterococcus*, *Weissella*, *Leuconostoc*) represented less than 2%. This could be related to the late lactation period. *Carnobacterium* was represented in two samples (2.1–3.8%). *Carnobacterium* has seldom been reported in human milk, although this bacterium could be a new, largely unexplored candidate for novel probiotic bacteria from human milk. One study reported that the presence of *Carnobacterium* in milk was associated with cesarean delivery [12], which is in agreement with the delivery procedure in the present study. It is believed that cesarean delivery influences the milk microbiota because of the differential exposure of the newborn to skin and environmental bacteria instead of vaginal microbiota and because the skin and oral cavity of newborns act as sources of colonization of the bacteria in breast milk [12].

Aerobic endosporeformers of the genera *Bacillus* and *Paenibacillus* also had high relative abundances in several milk samples and contributed (together with *Streptococcus*) to the increase of *Firmicutes* during late lactation. Remarkably, genus *Bacillus* reached ca. 25% relative abundance in two samples (weeks 16 and 27). The presence of *Bacillus* in breast milk samples has also been reported in several previous studies [21,25,26,27,28]. Patel et al. [28] reported both the presence of *Bacillus* and *Paenibacillus* in human milk, but *Bacillus* was associated with subacute or acute mastitis. However, during the sampling period of the present study, the mother did not report any signs of mastitis. Members of genus *Bacillus* are ubiquitous, sporulating, saprophytic microorganisms that can readily contaminate human milk during its collection or storage [29]. Among them, *B. cereus* is a matter of concern in stored breast milk (specially in breast milk banks) since it can produce food poisoning toxins and may also cause severe illness in neonates [29,30,31]. On the other hand, selected strains of *Bacillus* species are commercialized as human probiotics [32] and strains of *Bacillus coaulans* and *Bacillus clausii* are being investigated for pediatric and infant formula applications [33,34,35].

Results from the present study indicate large variations in the microbiota of the breast milk from a single woman during the sampling period. Tentatively, some of the changes could be explained by environmental contamination of the milk. The baby was already in the weaning period and crawling. Therefore, transfer of microbiota from the baby to the mother’s breast could account for some of the contaminants observed, including Enterobacteria. Previous work has suggested that the environment, utensils, and water could contribute considerably to the microbiota detected in breast milk [6]. A second way of contamination could be during milk expression by the breast pump. It has been reported that breast pumps may be difficult to decontaminate and increase the bacterial contamination of the milk [36]. And, finally, contamination could also occur during sample processing and analysis operations. Additionally, previous studies reported that contaminant DNA (sample collection and preparation, laboratory environment and reagents, personnel…) and cross contamination (e.g., during sequencing runs) can influence the results of next-generation sequencing approaches [37,38]. However, none of the variations reported in the present study were observed in other studies carried out in the same lab and using the same reagents and therefore it seems unlikely that they may be due to lab contamination. In addition, the negative controls included in the Illumina amplification and sequencing protocol clearly ruled out the possibility of contamination at this step.

## 5. Conclusions

In conclusion, results from the present study reveal major changes in the microbiota of breast milk from a single mother during the late breastfeeding period. The main changes include a decrease in the relative abundance of *Staphylococcus* and an increase of other microbial groups belonging to phylum *Proteobacteria*. Factors such as environmental contamination and changes in the baby habits (weaning and crawling) could account for the observed differences during the sampling period.

## Figures and Tables

**Table 1:** N° of reads and alpha diversity indexes at genus level of breast milk samples at different weeks (W) of the sampling period.

Sample	N° Reads	N° Observations	Chao1	Shannon-Weaver	Simpson
W1	111,968.00	107.00	107.00	2.90	0.89
W2	64,302.00	91.00	91.0	3.32	0.94
W3	106,190.00	95.00	95.00	1.60	0.52
W4	125,198.00	165.00	165.00	3.37	0.93
W5	145,841.00	115.00	115.00	1.87	0.70
W6	132,594.00	71.00	71.00	2.08	0.83
W7	145,004.00	163.00	163.00	2.89	0.84
W8	122,052.00	65.00	65.00	1.24	0.47
W9	151,193.00	110.00	110.00	2.94	0.89
W10	86,107.00	140.00	140.00	3.49	0.94
W11	3245.00	9.00	9.00	1.34	0.66
W12	55,527.00	65.00	65.00	2.02	0.78
W13	138,427.00	118.00	118.00	2.27	0.81
W14	141,003.00	93.00	93.00	1.86	0.65
W15	143.00	3.00	3.00	0.32	0.14
W16	102,600.00	102.00	102.00	2.45	0.80
W17	98,764.00	81.00	81.00	2.00	0.68
W18	107,346.00	1370.00	130.00	3.31	0.92
W19	140.00	3.00	3.00	0.62	0.37
W20	68,575.00	90.00	90.00	2.69	0.85
W21	1,375,641.00	119.00	119.00	1.92	0.68
W22	89,705.00	92.00	92.00	1.65	0.58
W23	116,236.00	104.00	104.00	1.82	0.62
W24	100,113.00	102.00	102.00	2.34	0.76
W25	97,925.00	109.00	109.00	2.52	0.86
W26	61,413.00	6.00	6.00	0.01	0.00
W27	105,221.00	119.00	119.00	2.72	0.87
W28	36,336.00	111.00	111.00	2.77	0.81
